# Prevention of High Glucose-Mediated EMT by Inhibition of Hsp70 Chaperone

**DOI:** 10.3390/ijms22136902

**Published:** 2021-06-27

**Authors:** Alina D. Nikotina, Snezhana A. Vladimirova, Elena Y. Komarova, Dmitry Alexeev, Sergey Efremov, Elizaveta Leonova, Rostislav Pavlov, Viktor G. Kartsev, Sergey G. Polonik, Boris A. Margulis, Irina V. Guzhova

**Affiliations:** 1 Institute of Cytology of Russian Academy of Sciences, Tikhoretsky Ave. 4, 194064 St. Petersburg, Russia; nikotina.ad@gmail.com (A.D.N.); snezhana.alexandrovna@mail.ru (S.A.V.); elpouta@yahoo.com (E.Y.K.); dm_aleksee@mail.ru (D.A.); margulis@incras.ru (B.A.M.); 2 Department of Anesthesiology and Intensive Care, Saint-Petersburg State University Hospital, Fontanka River enb.154, 190103 St. Petersburg, Russia; sergefremov@mail.ru (S.E.); eliz.leonova@gmail.com (E.L.); rostislavpavlov777@gmail.com (R.P.); 3 InterBioScreen, Institutsky Ave. 7a, Chernogolovka, 142432 Moscow, Russia; vkartsev@ibscreen.chg.ru; 4 G.B.Elyakov Pacific Institute of Bioorganic Chemistry of Far East Branch of Russian Academy of Sciences, Prospect 100 let Vladivostoku, 159, 690022 Vladivostok, Russia; sergpol@piboc.dvo.ru

**Keywords:** colon cancer, hyperglycemia, EMT, Hsp70, CL-43, PES

## Abstract

Hyperglycemia may contribute to the progression of carcinomas by triggering epithelial-to-mesenchymal transition (EMT). Some proteostasis systems are involved in metastasis; in this paper, we sought to explore the mechanism of Hsp70 chaperone in EMT. We showed that knockdown of Hsp70 reduced cell migration capacity concomitantly with levels of mRNA of the Slug, Snail, and Twist markers of EMT, in colon cancer cells incubated in high glucose medium. Conversely, treatment of cells with Hsp70 inducer U-133 were found to elevate cell motility, along with the other EMT markers. To prove that inhibiting Hsp70 may reduce EMT efficiency, we treated cells with a CL-43 inhibitor of the HSF1 transcription factor, which lowered Hsp70 and HSF1 content in the control and induced EMT in carcinoma cells. Importantly, CL-43 reduced migration capacity, EMT-linked transcription factors, and increased content of epithelial marker E-cadherin in colon cancer cells of three lines, including one derived from a clinical sample. To prove that Hsp70 chaperone should be targeted when inhibiting the EMT pathway, we treated cancer cells with 2-phenylethynesulfonamide (PES) and demonstrated that the compound inhibited substrate-binding capacity of Hsp70. Furthermore, PES suppressed EMT features, cell motility, and expression of specific transcription factors. In conclusion, the Hsp70 chaperone machine efficiently protects mechanisms of the EMT, and the safe inhibitors of the chaperone are needed to hamper metastasis at its initial stage.

## 1. Introduction

Colorectal cancer is currently the third most common cancer worldwide and the second most fatal one [1,2]. The extremely high mortality of patients with this type of cancer is due to the increased metastatic activity of cancer cells [3], associated with their phenotypic conversion, known as epithelial-to-mesenchymal transition (EMT) [4]. During this process, epithelial cells activate a program to remodel their organization and achieve a mesenchymal fate. EMT is controlled by a few transcription factors (Snail, Slug, and Twist) regulating the expression of E-cadherin and vimentin [5].

Hyperglycemia is just one factor that contributes to the various types of oncological pathologies, including colorectal cancer, which is usually linked to diabetes. According to Warburg, elevated glucose levels are associated with tumorigenesis [6]. This interrelationship was proven when studying patients with diabetes who had dramatically elevated risks of developing cancer, including colorectal cancer [7]. Of note, it was recently demonstrated that hyperglycemia induced TGF-β secretion in human lung cancer cells A549 [8], leading to EMT [9]. High glucose also promoted breast cancer cell invasion by inducing EMT [10,11,12]. The sequence of events, comprising high glucose, EMT, increased resistance and mobility of tumor cells, metastasis and, finally, massive cell death, is well documented. A variety of methods has been developed to break this deadly chain, including special diets that should work on the upstream stage of the above process [13]. Testing inhibitors of proteostasis (e.g., of molecular chaperones) as factors that disarm cell protection mechanism, based on Hsp70 chaperone, appears promising.

Hsp70 (HSPA1A) is often overexpressed in cancer cells, correlating with aggressiveness and poor prognosis for patients [14]. Chaperone Hsp70 binds proteins taking part in almost all known processes of cell physiology and, therefore, interferes with apoptosis pathways [15,16,17], or the cell growth process [18,19], leading to cancer cells resisting anti-tumor therapy. Elevated Hsp70 expression has been shown to correlate with lymph node metastasis in breast cancer [20] and with vascular invasion of gastric cancer cells [21]. Concomitantly, several reports have demonstrated that Hsp70 is able to inhibit TGF-β by Smad2 phosphorylation and decrease EMT [22,23]. The aim of this study was to explore the role of Hsp70 in EMT, in human colon cancer cells, in hyperglycemic conditions.

## 2. Results

### 2.1. Expression of Hsp70 Links to the Capacity of Hyperglycemia-Induced EMT

**Figure 1:**
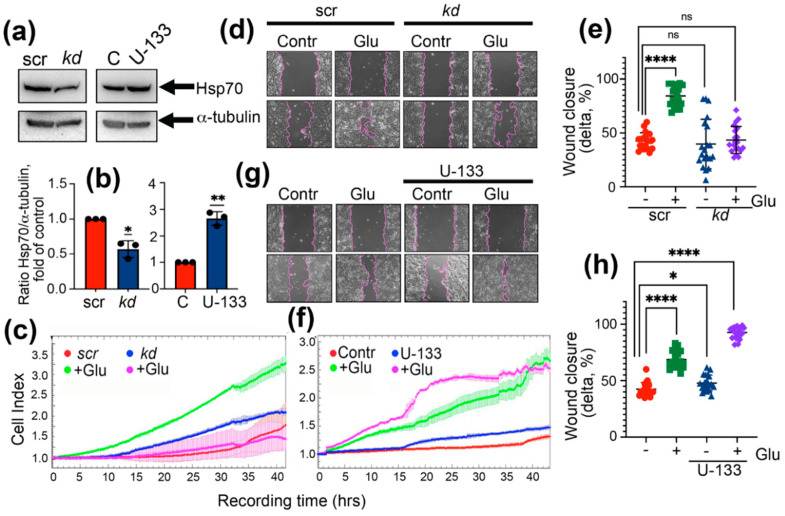
DLD1 cell motility in hyperglycemic conditions depends on Hsp70 level. (**a**) Western blot of DLD1 (*scr*, *kdHsp70*, and control DLD cells induced with 3 μM U-133 with antibody against Hsp70). Antibodies to α-tubulin were used to specify loading control Representative data of three independent experiments are presented. (**b**) Ratio of the intensity of the Hsp70 bands to the intensity of the α-tubulin bands. * *p* = 0.0148, ** *p* = 0.0031. (**c**) DLD1cells, *scr* and *kdHsp70 (kd)* were incubated with 80 mM glucose over 6 days, seeded to 16-well CIM plates and then monitored with the aid of xCELLigence equipment over 44 h. As a control, the migration characteristics of DLD1*scr* and DLD1*kd* without incubation with glucose were used. (**d**) DLD1*scr* and DLD1*kd* cells with or without incubation in the presence of high glucose were cultured in 6-well plates. A straight scratch was made in individual wells with a 1000 μL pipette tip, and the wound was photographed 24 h later. (**e**) The wound closure was calculated as the ratio of the distance of the overgrown area of the scratch at 24 h to the distance at 0 h after damage in 21 lines (delta). The distance was measured using the Adobe Photoshop CS6 program. Data are presented in % of delta. **** *p* < 0.0001. (**f**) DLD1 cells were incubated with 80 mM of glucose and 3 μM of U-133 over 6 days and then were monitored with the aid of xCELLigence equipment over 44 h. (**g**) Wound assay of DLD1 cells induced with 80 mM of glucose and 3 μM of U-133. (**h**) The wound closure was calculated as on (**e**). * *p* = 0.0124, **** *p* < 0.0001.

To test whether Hsp70 affects the motility of colon cancer cells, we used DLD1 cells with downregulated Hsp70 and those treated with U-133, a compound previously found to induce the heat shock response [24,25]. Western blotting data showed that, in DLD1*kd* cells, the chaperone level was reduced to 57 ± 0.1 and after induction with U-133, the level of Hsp70 was increased approximately 3-fold (Figure 1a,b).

We measured the migration capacity using two approaches: with the aid of CIM plates and xCELLigence equipment and using a wound-healing assay. According to xCELLigence data, untreated DLD1 cells showed weak capacity to migrate through the pores of CIM plates (Figure 1c), whereas the wound-healing assay showed that the wound was healed by 42.5 ± 1.7% over 24 h (Figure 1d,e). The cell index of DLD1*kd* cells identified in xCELLigence data reached a value of 1.45 after 40 h of recording (Figure 1b); however, the wound was healed only by 38.7 ± 5.2% (Figure 1d,e). In the presence of a high content of glucose, DLD1*scr* cells started to move through the pores at 3–4 h after the recording was started, and in the end, the cell index was equal to 3.3 (Figure 1c) that nicely correlated with the wound closure assay where the healing reached 84.3 ± 2.0% (Figure 1d,e). Knockdown of Hsp70 led to a reduction of the wound healing to 43.4 ± 2.8% and fully abolished the ability of cells to migrate through the pores, as was shown in xCELLigence data (Figure 1c–e). Elevation of Hsp70 synthesis with U-133 also led to an increase of DLD1 cell motility in both assays. The cell index corresponding to migrating cells reached the value 1.3 (xCELLigence test) and the wound closed a little faster (47.9 ± 1.7% vs. 42.5 ± 1.3% in untreated DLD1 cells), but in the presence of 80 mM glucose, the treatment with U-133 increased the migration of DLD1 cells in both assays. The cell index was 2.5 vs. 1.8 in untreated cells after 20 h of recording and wound closure reached 92.1 ± 1.2% in these cells compared to 68.7 ± 1.7% in DLD1 cells treated with high glucose (Figure 1f–h).

**Figure 2:**
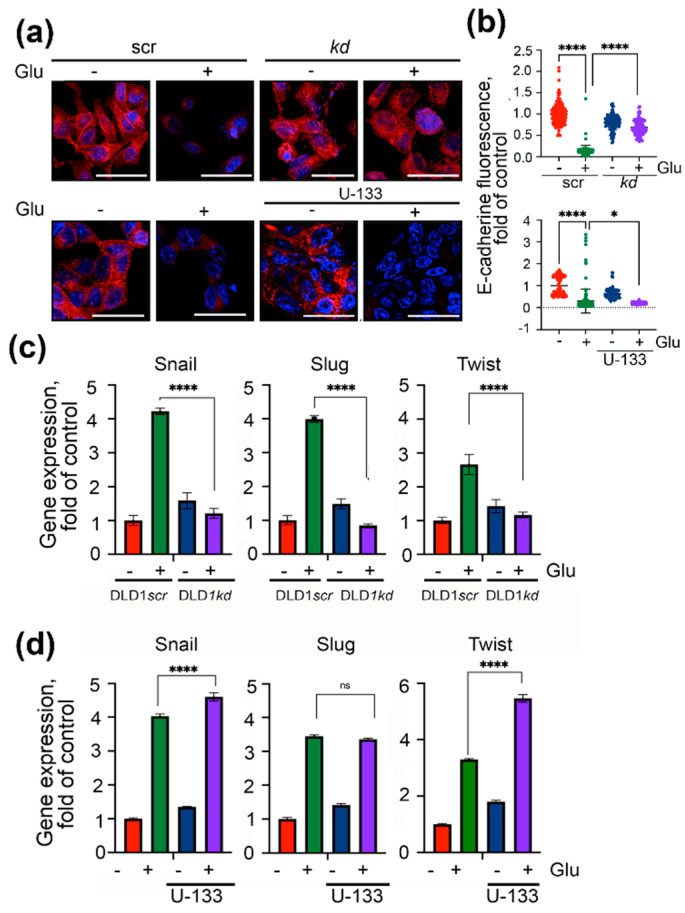
Expression of EMT markers in DLD1 cells cultured in a high glucose concentration depends on Hsp70 content. (**a**) E-cadherin expression in DLD1*scr* and DLD1*kd* cells incubated and not incubated in 80 mM glucose (upper line) and DLD cells treated with 3 μM U-133 and 80 mM glucose (lower line) were revealed with aid of confocal microscopy. Scale bar 10 μm. (**b**) The fluorescence of each single cell was measured with the aid of Image J. Data are presented as a fold of control. * *p* = 0.0350, **** *p* < 0.0001. (**c**,**d**) Snail, Slug and Twist gene expression in cells with various contents of Hsp70 was revealed with qPCR. **** *p* < 0.0001.

The expression of EMT markers in DLD1 cells, control cells, and cells with reduced Hsp70 content correlated well with the data of migration tests. We stained the DLD1 cells, untreated, *kd* cells with downregulated Hsp70 and DLD1 cells after incubation with U-133 with antibody against E-cadherin, and found that, in untreated DLD1 and DLD1*scr* cells, E-cadherin was expressed at high levels and a part of its molecules appeared on the cell’s surface. The high glucose in the culture medium led to a decrease in the total amount of E-cadherin in cells to 14.3 ± 1.1% and its escape from the plasma membrane. We observed a slight decrease in the expression of E-cadherin in DLD1 cells treated with U-133 to 64.0 ± 1.8%. However, treatment with U-133 in the presence of high glucose led to full disappearance of E-cadherin in DLD1 cells (9.1 ± 0.03%), whereas the E-cadherin pattern in DLD1*kd* cells in the same conditions did not differ from that in untreated DLD1or DLD1*scr* cells (Figure 2a,b).

The levels of Snail, Slug, and Twist in DLD1 cells in normal conditions or with elevated glucose were evaluated with the aid of qPCR. The hyperglycemia of cells with Hsp70 knockdown led to downregulation of Snail by 3.5-fold, Slug by 4.8-fold, and Twist by 2.3-fold, as compared with those in DLD1*scr* cells (Figure 2c). Elevation of Hsp70 as a result of treatment with U-133 in DLD1 cells in the presence of high glucose increased Snail gene expression by 14.3%, did not change Slug expression, and increased TWIST expression by 1.7-fold (Figure 2d).

### 2.2. Suppressing Hsp70 Reduces the Ability of Colon Cancer Cells to Pass EMT under Hyperglycemia Conditions

**Figure 3:**
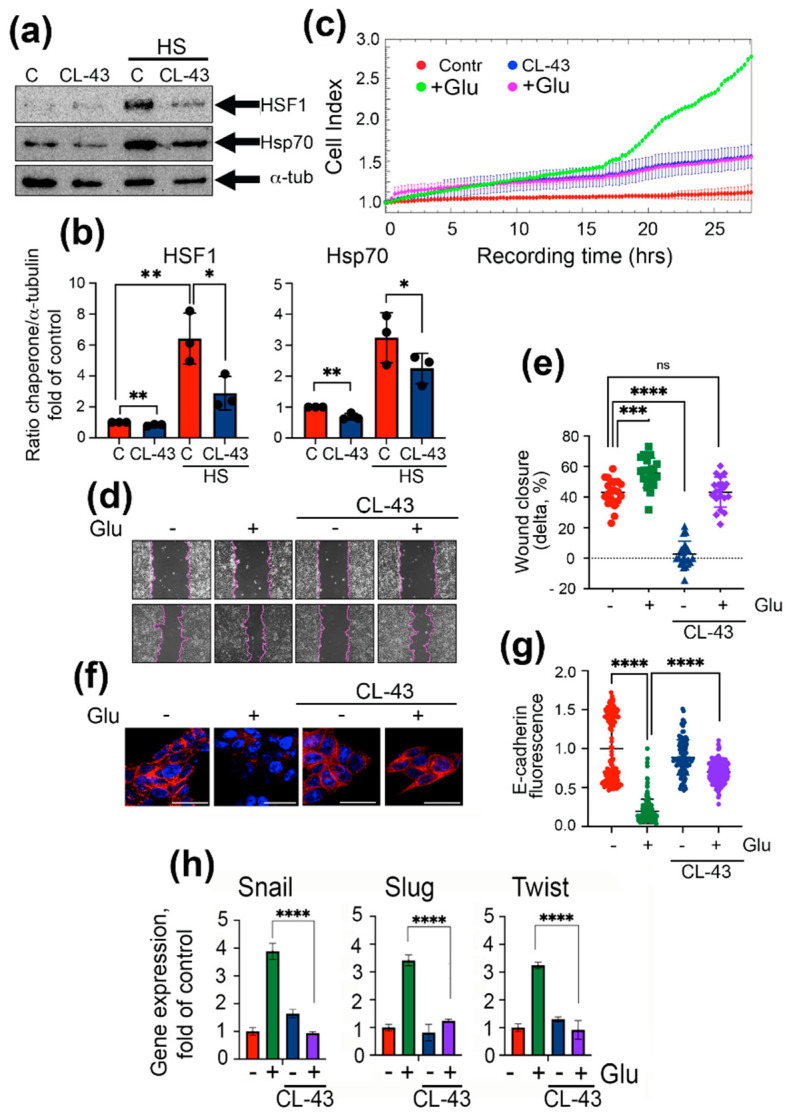
Inhibitor of master regulator HSF1 (CL-43) decreases cell motility and EMT markers expression in DLD1 cells. (**a**) Western blotting of DLD1 cells, controls and cells treated with 500 nM CL-43 before heat shock (43 °C, 30 min, and 18 h of recovery) probed with antibody against HSF1, Hsp70 and α-tubulin (as a loading control). (**b**) Ratio of intensity of HSF1 or Hsp70 bands to band intensity of a-tubulin. Band intensity was measured with the aid of ImageJ. Data of three western blots were calculated. * *p* = 0.0212, 0.0442, ** *p* = 0.0020, 0.0080, 0.0015. (**c**) After 6 days of incubation with 80 mM glucose and 0.5 μM CL-43, DLD1 cells were seeded to 16-well CIM plates and then monitored with the aid of xCELLigence equipment over 28 h. (**d**) Wound healing assay of DLD1 cells treated as in (**b**) with calculation (**e**) as the ratio of the distance of the overgrown area of the scratch at 24 h to the distance at 0 h after damage in 21 lines (delta). The distance was measured using the Adobe Photoshop CS6 program. Data are presented in % of delta. *** *p* = 0.0001, **** *p* < 0.0001 (**f**) E-cadherin expression in DLD1 cells treated with 80 mM glucose and 500 nM CL-43 revealed with the aid of confocal microscopy. (**g**) The fluorescence of each single cell was measured with the aid of Image J. Data are presented as a fold of control. **** *p* < 0.0001. (**h**) Snail, Slug, and Twist gene expression in DLD1 cells treated over 7 days with 80 mM glucose and 500 nM CL-43 analyzed with the aid of qPCR.

The major goal of the present work was to study the inhibitors of Hsp70 synthesis and function. One such inhibitor, CL-43, was found to reduce the activity of the HSF1 master transcription factor and to decrease the content of heat shock proteins in a variety of tumor cell types [26], and we confirmed these effects in DLD1 cells. First, we proved that CL-43 causes a reduction of HSF1 and Hsp70 content, and to check whether it is true for cells with an already stimulated heat shock response mechanism, we used control DLD1 cells and cells subjected to heat shock (HS) at 43 °C (30 min) 2 h after treating them with CL-43 at a concentration of 500 nM. Western blotting was performed 18 h after induction. We observed that CL-43 reduced the amount of both HSF1 and Hsp70, while the suppression was more pronounced in heat-stressed cells (Figure 3a,b). Interestingly, we noticed that HS increased the accumulation of HSF1 approximately 6-fold, which was revealed using antibodies detecting total HSF1, but CL-43 reduced its amount by 2-fold. Inhibition of HSF1 caused a reduction of Hsp70 content in heat-treated cells by 30% (Figure 3a,b).

The analysis of cell motility performed with the aid of xCELLigence and the wound healing assay demonstrated an effect similar to that shown earlier for untreated DLD1 and DLD1*kd* cells. Using xCELLigence data, we observed the weak elevation of migration capacity under CL-43 (Figure 3c); however, wound closure over 24 h was slower than in untreated (control) cells (2.8 ± 1.8% vs. 43.1 ± 1.9%). Elevation of migration under high glucose conditions was shown with the aid of both tests, as well as inhibition of motility due to CL-43 administration (Figure 3c–e).

Using confocal microscopy, we found that the expression and localization of the epithelial marker E-cadherin in DLD1 cells under hyperglycemic conditions was almost fully suppressed, while CL-43 reversed that pro-EMT action (Figure 3f,g). The expression of other EMT markers (i.e., Snail, Slug, and Twist) assessed by qPCR also decreased down to the control level when CL-43 was used in the presence of a high glucose concentration (Figure 3h).

**Figure 4:**
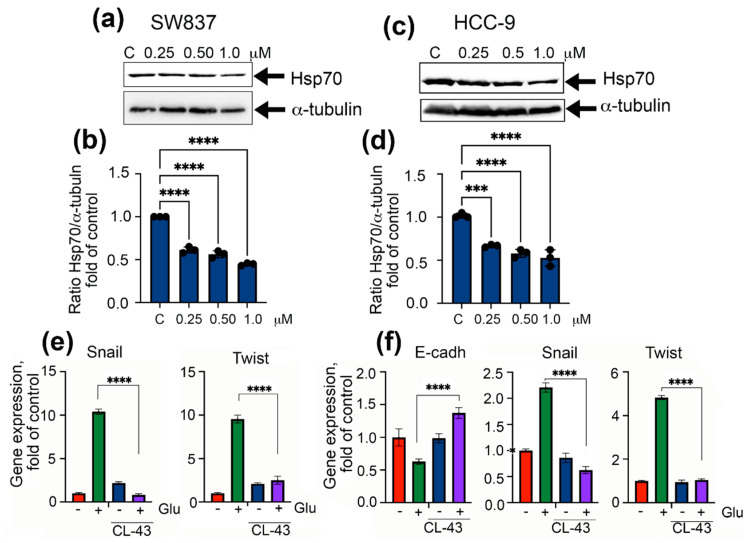
CL-43 is able to reduce expression of EMT markers in other colon cancer cell lines, including freshly isolated colon cancer cells from patients. (**a**) Western blotting of SW837 cells treated with 0.25, 0.5, and 1.0 μM of CL-43 with antibody to Hsp70 and (**b**) ratio of intensity of Hsp70 bands to band intensity of a-tubulin. Band intensity was measured with the aid of ImageJ. Data of three western blots were calculated. **** *p* < 0.0001. (**c**) Western blotting of HCC-9 cells and (**d**) ratio of intensity of Hsp70 bands to band intensity of a-tubulin. *** *p* = 0002, **** *p* < 0.0001. (**e**) Snail and Twist expression in SW837 cells after 7 days of incubation with 500 nM CL-43 and 80 mM glucose. **** *p* < 0.0001. (**f**) E-cadherin, Snail, and Twist expression in HCC-9 cells cultured in the presence of 500 nM CL-43 and 80 mM glucose. **** *p* < 0.0001.

Similar results were obtained when we employed two other colorectal cancer cell lines, SW837 and HCC-9 isolated from the colon tumor. First, we found that the Hsp70 level was dose-dependently reduced in both cell lines, reaching 44.7 ± 0.2% and 52.7 ± 0.4% of that in untreated cells (C) when CL-43 was administered in a maximal concentration (Figure 4a–d). As expected, high glucose greatly elevated the expression of EMT markers (i.e., Snail and Twist), while application of CL-43 lowered them by 12-fold and 4-fold in SW837 and HCC-9 cells, respectively (Figure 4e,f). In HCC-9 cells, incubation in hyperglycemic conditions caused the reduction of the E-cadherin level, while using CL-43 significantly elevated its expression, thus suggesting the drug-mediated suppression of the EMT process (Figure 4f).

### 2.3. Inhibition of Chaperonic Function Leads to Downregulation of EMT in Colon Cancer Cells

The cytoprotective activity of Hsp70 is commonly thought to be linked to its ability to recognize damaged proteins and convert them into active forms, or to target the incorrigible polypeptides to proteasomal degradation with a few protein aides. To disrupt this chaperonic mechanism, a few small molecules have been developed. We chose PES, because of its ability to interact with the substrate-binding domain of Hsp70, which harbors the chaperone protein targets [27] and is known to possess autonomous anti-cancer activity [28].

**Figure 5:**
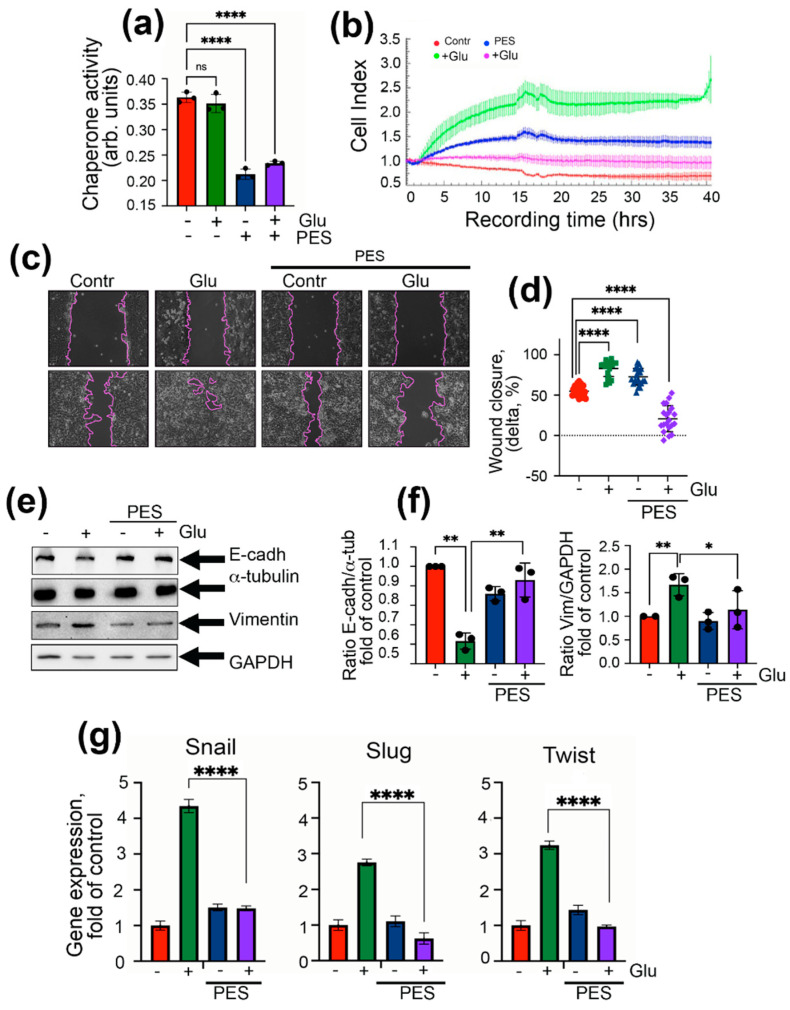
Inhibitor of Hsp70 function, PES (or pifithrin-μ), reduces EMT in DLD1 cells in high glucose conditions. (**a**) Data of chaperone activity assay of cells treated with 3 μM PES and 80 mM glucose. **** *p* < 0.0001. (**b**) After 6 days of incubation with 80 mM glucose and 3 μM PES, DLD1 cells were seeded to 16-well CIM plates, and were then monitored with the aid of xCELLigence equipment over 40 h. (**c**) Wound healing assay of DLD1 cells treated as in (**d**) with calculation of % of delta between distance of cells at 0 and 24 h **** *p* < 0.0001. (**e**) Western blot of DLD1 cells treated with 3 μM PES and 80 mM glucose probed with antibodies against E-cadherin and vimentin and (**f**) calculation of ratio of E-cadherin band intensity to a-tubulin band intercity as fold of control (left panel) and ratio of vimentin band intensity to GAPDH band intensity (right panel). ** *p* = 0.0015, 0.0029, 0.0065; * *p* = 0.0393. (**g**) qPCR data of Snail, Slug, and Twist in DLD1 cells treated with 3 μM PES and 80 mM glucose. **** *p* < 0.0001.

To check whether PES inhibits chaperonic activity in DLD1 cells, we employed a substrate-binding assay in which the cellular Hsp70 chaperone interacts with CMLA; this binding occurs in the first stage of the chaperonic process [24]. PES was found to reduce the chaperonic power of Hsp70 by 35.6%, both in normal and in high glucose medium conditions, proving the anti-chaperonic activity of the compound (Figure 5a). As before, we measured migration capacity using two assays; in the xCELLigence two-chamber assay, PES suppressed the migration properties of cells under hyperglycemic conditions, which was revealed using both tests (Figure 5b–d). The results of the wound healing assay confirmed the above data; interestingly, in hyperglycemic cells, PES inhibited migration capacity to the 20.8 ± 3.6% (Figure 5c,d). The level of E-cadherin measured with the aid of Western blotting was reduced to 61.7 ± 2.4% in cells grown in the presence of high glucose, whereas the vimentin level, on the contrary, was elevated by 67%, which is typical for the EMT profiles of these markers. Administration of PES led to the elevation of E-cadherin content and downregulation of vimentin in high glucose conditions (Figure 5e,f). The levels of three EMT markers were also downregulated when PES was used in high glucose conditions (Figure 5g).

**Figure 6:**
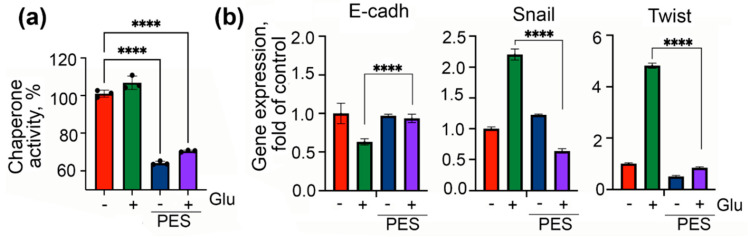
Inhibitor of Hsp70 function, PES, suppresses expression of EMT markers in patient’s HCC-9 cells**.** (**a**) HCC-9 cells were incubated with 3 μM PES and 80 mM glucose over 6 days and the chaperone activity of Hsp70 expressed in these cells was measured with aid of Chap-ELISA. **** *p* < 0.0001. (**b**) Levels of E-cadherin, Snail, and Twist in HCC-9 cells treated with PES and high glucose were measured with aid of qPCR.

We obtained similar results using HCC-9 cells, which were freshly isolated from patients. PES decreased the ability of the chaperone to bind denatured protein by 38.2% in these cells (Figure 6a). It also restored the E-cadherin expression and reduced Snail and Twist expression in hyperglycemic conditions (Figure 6b).

Taken together, these data demonstrate that, irrespective of how we decreased Hsp70 expression, genetically or chemically, or disturb its functional activity, EMT caused by high glucose content was not implemented.

## 3. Materials and Methods

### 3.1. Cells and Treatment

Human colorectal adenocarcinoma cells DLD1 and SW837 were obtained from the Russian Collection of Cell Cultures (Institute of Cytology of Russian Academy of Sciences, St. Petersburg, Russia). HCC-9 cells were obtained from biopsy material from a 56-year-old female patient from St. Petersburg State University N.I. Pirogov Clinic of High Medical Technologies, with a diagnosis of colon cancer of the hepatic bend pT3N2b (7/19) M0, G2, PN -, VI -, and III stage [29]. The study was approved by the local ethics committee of St. Petersburg State University Hospital (St. Petersburg, Russia). The written informed consent of the patient was obtained according to the Declaration of Helsinki [30]. On the same day that the biopsy was performed, the tumor tissue was mechanically disintegrated, and the cell suspension was seeded to a 12-well plate (TPP, Trasadingen, Switzerland). The cells had three passages, and they were frozen in liquid nitrogen. For the experiments, HCC-9 cells were thawed and multiplied in (at least) two passages. All cells used in this work were maintained in Dulbecco’s Modified Eagle Medium, containing 25 mM glucose (Thermo Fisher, catalogue number 41965039, Thermo Fisher, Waltham, MA, USA), 10% fetal calf serum (catalogue number A3161001, Thermo Fisher, Waltham, MA, USA), 100 units/mL penicillin, and 0.1 µg/mL streptomycin (Biolot, Saint Petersburg, Russia) under 5% CO_2_ at 37 °C.

The plasmids for knockdown of Hsp70 (HSPA1A) contained the following sequences, antisense TTGATGCTCTTGTTCAGGTCG, and scrambled RNA, TAATACGACTCACTATAGGG; they were obtained from Evrogen (Moscow, Russia). Packaging (psPAX2) and envelope (pMD2.G) plasmids was generously provided by Dr. A. Tomilin (Institute of Cytology of Russian Academy of Science, Saint Petersburg, Russia). To transfect HEK-293T cells, we employed polyethylenimine and a mixture of three plasmids; selection was performed with the aid of 2.0 μg/mL puromycin (Sigma-Aldrich, St. Louis, MO, USA) for at least 2 weeks before the experiment. To get DLD1scr cells, DLD1 cells were infected with a lentivirus with the gfp gene. To induce EMT, D-glucose (Sigma-Aldrich, St. Louis, MO, Germany) was added to the cell culture up to a final concentration of 80 mM. Afterwards, the cells were left for 6 days before analysis.

A cell migration assay was performed with the aid of xCELLigence Real-Time Cell Analyzer DC equipment (Agilent, Santa Clara, CA, USA). This impedance-based assay carries out label-free, real-time, high-throughput analysis of cell growth and migration. After 6 days of incubation with glucose and Hsp70 modulators, DLD1 cells were placed on a 16-well CIM plate for migration analysis, and monitored for the next 25–44 h; data computation was performed using RTCA Analysis software.

### 3.2. Wound Healing Assay

DLD1 cells were cultivated in a 6-well plate (4.5 × 10^5^ cells/well) in the presence of a high concentration of glucose, with or without Hsp70 modulating substances, for 6 days. In a monolayer that reached 85% confluence, a scratch was created using a 1000 μL dispenser tip. The monolayer cells were photographed immediately after applying the scratch and after 24 h. The distance between the cells was calculated with the aid of PhotoShopCS6.

### 3.3. Chemicals

For induction and inhibition of Hsp70 expression or function, certain chemicals were utilized, including the HSF1 inducer U-133, which was previously selected from the library of small molecules of the Pacific Institute of Bioorganic Chemistry, Russian Academy of Sciences (Vladivostok, Russia). U-133 is an acetylated tris-O-glucoside echinochrome and it was obtained by the chemical modification of the sea urchins pigment echinochrome [31]. U-133 was found to induce synthesis in human U-937 promonocytes [24] and in the substantia nigra of rats [25]. The HSF1 inhibitor CL-43 was previously shown to inhibit Hsp70 synthesis in various cancer cells [26]. Moreover, 2-phenylethynesulfonamide (PES) or pifithrin-μ, an inhibitor of the chaperonic function of Hsp70 [32], was purchased at Sigma-Aldrich (St. Louis, MO, USA).

### 3.4. Western Blotting

Colon cancer cells treated with chemicals were lysed on ice in a solution containing 20 mM Tris-HCl pH 7.5, 150 mM NaCl, 2 mM EDTA, 1 mM PMSF, 0.5% sodium deoxycholate, 0.1% SDS, 0.1% Triton X-100, and a protease inhibitor cocktail (catalogue number P8340, Sigma-Aldrich, St. Louis, MO, USA). After the total protein concentration was measured (Bradford, 1980), the supernatants were precipitated with ice-cold acetone for 1 h at −20 °C. Sedimented proteins were dissolved in a sample buffer containing 2% SDS, 10% 2-mercaptoethanol, 20% glycerol, 0.004% bromophenol blue, and 0.125 M Tris-HCl pH 6.8. Equal amounts of proteins were electrophoresed in 11.5% polyacrylamide gel and then transferred onto a PVDF membrane using a Pierce Power Station (Thermo Fisher, Waltham, MA, USA). The membrane was blocked with phosphate-buffered saline (PBS) containing 5% (*w*/*v*) skimmed milk and incubated with primary and secondary antibodies (Table 1) at room temperature for 1 h. Images were captured by a ChemiDoc (Bio-Rad, Hercules, CA, US) detection system with Femto ECL reagent (catalogue number 34096, Thermo Fisher, Waltham, MA, USA). The antibodies used in the study are represented in Table 1.

### 3.5. RNA Isolation and Quantitative Real-Time PCR

RNA was isolated using TRIzol (Thermo Fisher, catalogue number 15596026, Waltham, MA, USA) and converted to DNA using a MMLV RT kit (Evrogen, Moscow, Russia), according to the manufacturer’s protocol. All RT-PCR studies were performed using a CFX96 Real-Time PCR detection system (Bio-Rad, Hercules, CA, USA) and qPCRmix-HS SYBR (Evrogen, Moscow, Russia), according to the manufacturer’s protocol; melt curve analysis was employed to prove amplicon accuracy. The data were analyzed for fold-change using Bio-Rad CFX software (version 3.1).

The sequences of the primers are represented in Table 2.

### 3.6. Immunocytochemistry

DLD1 cells were cultivated in the presence of a high concentration of glucose, with or without Hsp70 modulating substances, for 6 days, and then seeded to cover glasses placed in a 24-well plate. Twenty-four hours later, cells were fixed with 4% paraformaldehyde for 15 min, and then permeabilized with 0.1% Triton X-100 in PBS. After washing with PBS, cells were preincubated in blocking solution (1% bovine serum albumin, BSA) in PBST for 1 h at room temperature), and then incubated with primary mouse antibody against E-cadherin (ab40772, Abcam, Cambridge, UK,), overnight, in a humid chamber. After rinsing with PBS, the cells were incubated with a second anti-mouse antibody labeled with Alexa 647 (Abcam, Cambridge, UK) for 1 h. Nuclei were stained with 4′,6-diamidino-2-phenolindole dihydrochloride (DAPI). Fluorescence images were captured by an Olympus FV3000 confocal microscope and analyzed with cellSens software.

### 3.7. Chap-ELISA

To estimate the effect of PES on Hsp70 chaperonic activity in DLD1 and HCC-9 cells, we used a substrate-binding assay as described earlier [24]. Briefly, CMLA (10 μg/mL in PBS) was applied on the surface of a 96-microplate. Non-specific binding was then eliminated by applying 5 mg/mL BSA in PBS. The extracts were obtained from DLD1 and HCC-9 cells incubated with PES for 6 days. Cells were lysed by freeze–thaw cycles in HEPES 20 mM pH 7.5, NaCl 100 mM, MgCl_2_ 1.5 mM, NP-40 0.0025%, 0.5 mM EDTA, 0.5 mM DTT, and 10% glycerol, and after centrifugation at 10,000× *g* for 15 min, supernatants were transferred into wells covered with CMLA for 2 h at 37 °C. Anti-Hsp70 antibody (2B11) was applied, followed by incubation with anti-rat IgG conjugated with peroxidase (Sigma-Aldrich, St. Louis, MO, USA). After the addition of tetramethylbenzene in a citrate buffer (pH 4.5) containing hydrogen peroxide, the intensity of the staining was measured with the aid of a Varioskan LUX multipurpose reader (Thermo Fisher, Waltham, MA, USA).

### 3.8. Statistics

All results are given as the mean ± standard error of the mean (SEM) and represent the data from a minimum of three independent measurements. General statistical analysis was performed with the use of the non-parametric Mann–Whitney U-test (Shapiro–Wilk method). The results that gave Gaussian distribution were statistically processed by the one-way ANOVA test followed by Tukey’s comparison test. Which was employed with the aid of GraphPad Prism 9.0.2 (GraphPad Software, Inc., San Diego, CA, USA). Differences were considered statistically significant at *p* < 0.05.

## 4. Discussion

EMT is a multiphasic process leading to an expansion of phenotypically altered tumor cells with elevated resistance to anti-cancer drugs and increased motility as the necessary properties of metastasizing tissue. Post-EMT cells are not genetically distinct from their parental cells, but their properties become closer to that of so-called dormant or persister cells, in what relates to their non-proliferative state, enhanced resistance to therapy and likeness to cancer stem cells [33,34]. These hallmarks of aggressiveness require special tools for therapy, so we chose the inhibitors of proteostasis as a basic life-guarding mechanism. This approach is not novel since inhibitors of proteasomes, autophagy, and the Hsp90 chaperone are already employed in clinics, but the idea of our research was to destroy the Hsp70-based chaperonic system in particular, which is known to regulate the most important processes in a tumor cell by binding occasionally (or not occasionally) a variety of protein targets [35]. Of note, this interactome includes the polypeptides distinct to that of Hsp90 clients; therefore, it was tempting to reveal the effects of Hsp70 inhibitors on EMT-passing cells particularly.

To measure one of the key characteristics of cells undergoing EMT (motility), we employed DLD1 colorectal cancer cells incubated in an excess of glucose. Hyperglycemia, as we have shown, increased the rate of DLD1 motility by more than 2-fold as found by measuring the cell index with the aid of xCELLigence equipment. Importantly, this increase was less noticeable in the same cells with downregulated expression of Hsp70. The effect of Hsp70-dependent motility was earlier reported by Gong et al. demonstrated that knockout of Hsp70 in mouse mammary tumor cells almost completely stopped their motion and correspondingly inhibited the tumor growth in vivo [36]. The measurement of cell motility using the wound healing assay gave similar results, which was a less pronounced rate of scratch closure by DLD1 cells with downregulated Hsp70.

In order to be convinced in the mobility-initiating effect of the chaperone, we employed the inducer of its expression, U-133, an echinochrome derivative [24]. As expected, this compound increased Hsp70 expression in DLD1 cells and promoted the motility of DLD1 cells that was measured with two approaches. The observation that the upregulation of Hsp70 in cancer cells may result in their increased ability to pass EMT is corroborated with data indicating that the enhancement of the chaperone in hepatic carcinoma cells by heat stress was able to induce EMT-like and stem-like phenotypic changes [37].

To validate the data of experiments with the above assays, we measured the expression of typical EMT markers and found that E-cadherin escaped from DLD1 cells in hyperglycemic conditions, but was restored in cells with reduced levels of Hsp70, but this “disappearance” was augmented by the Hsp70 expression inducer U-133. As such, the recently discovered anti-tumor compound salidroside was found to increase the E-cadherin/N-cadherin ratio and inhibit proliferation, migration, and invasion of gastric cancer cells by downregulating the Src-associated signaling pathway and Hsp70 expression [38]. We also checked the expression of basal transcription factors activating EMT-associated phenotypic conversion and found that the expressions of Twist, Slug, and Snail were strongly elevated in DLD1 cells cultured in high glucose conditions; most surprising was that their mRNA content was increased even more in cells treated with U-133.

Analyzing data on the effects of Hsp70 regulation on EMT, one may be confused by their inconsistency in that the suppression of Hsp70 can either inhibit or promote phenotypic conversion of cancer cells before switching to metastasis. The data of Kasioumi et al. show that silencing of Hsp70 in HeLa, A549, and MCF7 cells caused considerable loss of motility, inability to form cadherin–catenin complexes, and stimulated their detachment from neighboring cells, which is the first step of anoikis and metastasis [39]. However, the link between the reduction of cell mobility and triggering of metastasis outlined by the authors is not clear. In a few studies, to trigger Hsp70 synthesis, geranylgeranylacetone has been employed, and it was found that overexpression of Hsp70 inhibited the TGF-β-induced EMT process and changed the cell morphology and migratory ability in A549 cells [40]. In an attempt to overcome such discordance, we chose hyperglycemia, which often accompanies the development of colorectal cancer in clinical practice [41], and found that the Hsp70 chaperone may harness the metastasis program by increasing cell motility and expression of three markers; this fact was laid in the basis of our model of EMT.

The major goal of the present work was to study how the inhibitors of Hsp70 synthesis and function may affect major characteristics of EMT. One such inhibitor, CL-43, was found earlier to reduce the activity of the HSF1 master transcription factor and to decrease the content of heat shock proteins in a variety of tumor cell types [25]. Estimating cell motility and the expression of major EMT markers, we found that, in the high glucose-mediated stimulation of metastasis, they could be efficiently suppressed by CL-43. Such effects were demonstrated with a few different HSF1 inhibitors, such as triptolide, KNK-437, KRIBB-11, and others [42]. One of the most potent HSF1 inhibitors, triptolide, was found to inhibit metastasis of B16 mouse melanoma cells [43], but its further pharmacological application is hampered because of toxicity issues; currently, newer triptolide derivatives being studied are exemplified by minnelide, but their function in EMT-mediated phenotypic conversion remains elusive [44]. Another HSF1 inhibitor, KRIBB-11, was found to reduce the motility and invasion of tumor cells in an orthotopic model of pancreatic cancer, proving the anti-EMT activity of the inhibitor of the heat shock response [45]. Earlier, CL-43 was found to upregulate the activity of well-established anti-tumor drugs, and to have low cytotoxicity [26]. The substance may become an ideal component of combinatorial schemes for the therapy of hyperglycemia-associated colon cancers.

The list of Hsp70 chaperone inhibitors includes JG-98, MKT-077, VER-155008 and others that, in most of experimental conditions, were demonstrated to reduce certain attributes of the EMT-metastasis pathway, migration capacity and modulation of appropriate markers, such as Slug, Twist1, Snail, vimentin, E/N-cadherin and others. We chose one of the most potent Hsp70 inhibitors, PES, and showed that it reduced the substrate-binding activity of the chaperone (e.g., prevented targeting of Hsp70 by its client proteins), whose list includes apoptosis cascade signaling molecules, regulators of intracellular transport, and autophagy [46]. These dissociations caused apparent changes in the EMT program as refer to migration capacity and to E-cadherin, Snail, and Twist expression. Notably, these changes also occurred in colon carcinoma cells taken from a patient. There are distinct views on the role of Hsp70 in EMT, particularly that triggered by TGF-β [23,47]. Therefore, our data prove the chaperone’s contribution to metastasis of hyperglycemia-associated tumors and, correspondingly, the search for efficient Hsp70 chaperone inhibitors, are actual. In conclusion, we demonstrated that, besides an active role in drug-associated apoptosis and participation in the formation of the tumor microenvironment, Hsp70 positively regulates the EMT process in hyperglycemia-coupled colon cancer, and novel medicines are necessary to diminish this chaperone capacity through a mono- or combination therapy technique [48].

## Figures and Tables

**Table 1:** The primary and secondary antibodies used in the study.

Antibodies to:	Manufacturer	Catalogue Number
Vimentin	Abcam	ab137321
E-cadherin	Abcam	ab40772
Hsp70	Homemade	clone 2E4
GAPDH	Thermo Fisher	AM4300
ß-tubulin	Thermo Fisher	MA1-80017
HSF1	Cell Signaling	#4356
GAM	Abcam	ab6789
GARabbit	Abcam	ab6721
GARat	Sigma-Aldrich (Merck)	AP136P
Goat pAb to Ms IgG Alexa647	Abcam	ab150115

**Table 2:** Sequences of primers used in the study.

β-actin	Forward	CCATCATGAAGTGTGACGTGG
	Reverse	GTCCGCCTAGAAGCATTTGCG
Snail	Forward	CATCCTTCTCACTGCCATG
	Reverse	GTCTTCATCAAAGTCCTGTGG
E-cadherin	Forward	AGGCCAAGCAGCAGTACATT
	Reverse	ATTCACATCCAGCACATCCA
Slug	Forward	ATGAGGAATCTGGCTGCTGT
	Reverse	CAGGAGAAAATGCCTTTGGA
Twist	Forward	TGCATGCATTCTCAAGAGGT
	Reverse	CTATGGTTTTGCAGGCCAGT

## Data Availability

HCC-9 cells and shHsp70 plasmid are available upon request.
